# Cultural Adaptation of Stress Management Intervention for Korean American Dementia Family Caregivers

**DOI:** 10.1093/geroni/igaf122.3566

**Published:** 2025-12-31

**Authors:** Hyejin Kim, Eunbee Liu, Olimpia Paun, Jaehyuk Song, Bruno Kajiyama, Seok Gyu Han, Dolores Gallagher-Thompson

**Affiliations:** Rush University, Chicago, Illinois, United States; Rush University, Chicago, Illinois, United States; Rush University, Chicago, Illinois, United States; Rush University, Chicago, Illinois, United States; Photozig, Inc., Moffett Field, California, United States; Rush University, Chicago, Illinois, United States; Stanford University, Stanford, California, United States

## Abstract

Korean American (KA) family caregivers of individuals with Alzheimer’s disease and related dementias (ADRD), particularly those caring for immigrants at home, face unique stress due to culturally specific care needs. We developed the Korean version of iCare (K-iCare), a culturally adapted, cognitive behavioral therapy (CBT)-based stress reduction intervention that has been widely adapted for various ethnic groups, but not for KAs. Guided by the Framework for Reporting Adaptations and Modifications-Enhanced (FRAME), K-iCare was developed through a five-stage process: (1) initial modification by the research team, (2) feedback from two focus groups, former KA ADRD caregivers (n = 5) and KA community service providers (n = 5), (3) refinement of K-iCare based on focus groups input, (4) validation by focus groups and the original iCare developers, and (5) finalization for implementation. Intervention materials were translated into Korean with careful cultural and linguistic adaptations. Terms without equivalent meaning in Korean were supplemented with both Korean and English wording for clarity. The total number of chapters was consolidated from nine to five, and caregiving scenarios were replaced with culturally relevant examples, such as emphasizing the traditional role of the eldest adult child in caregiving responsibilities. The intervention format was revised from self-paced asynchronous modules to synchronous weekly small-group sessions delivered in a hybrid format. A bilingual and bicultural doctoral-level nurse scientist with expertise in ADRD care will serve as the interventionist. Supporting materials (e.g., fidelity monitoring checklist) were developed to ensure consistent delivery. The next phase will evaluate the feasibility, acceptability, and preliminary efficacy of K-iCare.

